# Nonstructural Protein NSs of Schmallenberg Virus Is Targeted to the Nucleolus and Induces Nucleolar Disorganization

**DOI:** 10.1128/JVI.01263-16

**Published:** 2016-12-16

**Authors:** Julie Gouzil, Aurore Fablet, Estelle Lara, Grégory Caignard, Marielle Cochet, Cindy Kundlacz, Massimo Palmarini, Mariana Varela, Emmanuel Breard, Corinne Sailleau, Cyril Viarouge, Muriel Coulpier, Stéphan Zientara, Damien Vitour

**Affiliations:** a ANSES, UMR1161 Virologie, Laboratory for Animal Health, Maisons-Alfort, France; b INRA, UMR1161, Maisons-Alfort, France; c Université Paris-Est, Ecole Nationale Vétérinaire d'Alfort, UMR1161 Virologie, Maisons-Alfort, France; d MRC-University of Glasgow Centre for Virus Research, Glasgow, United Kingdom; University of Iowa

**Keywords:** Cellular shutoff, NSs, Schmallenberg virus, bunyavirus, nucleolus

## Abstract

Schmallenberg virus (SBV) was discovered in Germany in late 2011 and then spread rapidly to many European countries. SBV is an orthobunyavirus that causes abortion and congenital abnormalities in ruminants. A virus-encoded nonstructural protein, termed NSs, is a major virulence factor of SBV, and it is known to promote the degradation of Rpb1, a subunit of the RNA polymerase II (Pol II) complex, and therefore hampers global cellular transcription. In this study, we found that NSs is mainly localized in the nucleus of infected cells and specifically appears to target the nucleolus through a nucleolar localization signal (NoLS) localized between residues 33 and 51 of the protein. NSs colocalizes with nucleolar markers such as B23 (nucleophosmin) and fibrillarin. We observed that in SBV-infected cells, B23 undergoes a nucleolus-to-nucleoplasm redistribution, evocative of virus-induced nucleolar disruption. In contrast, the nucleolar pattern of B23 was unchanged upon infection with an SBV recombinant mutant with NSs lacking the NoLS motif (SBVΔNoLS). Interestingly, unlike wild-type SBV, the inhibitory activity of SBVΔNoLS toward RNA Pol II transcription is impaired. Overall, our results suggest that a putative link exists between NSs-induced nucleolar disruption and its inhibitory function on cellular transcription, which consequently precludes the cellular antiviral response and/or induces cell death.

**IMPORTANCE** Schmallenberg virus (SBV) is an emerging arbovirus of ruminants that spread in Europe between 2011 and 2013. SBV induces fetal abnormalities during gestation, with the central nervous system being one of the most affected organs. The virus-encoded NSs protein acts as a virulence factor by impairing host cell transcription. Here, we show that NSs contains a nucleolar localization signal (NoLS) and induces disorganization of the nucleolus. The NoLS motif in the SBV NSs is absolutely necessary for virus-induced inhibition of cellular transcription. To our knowledge, this is the first report of nucleolar functions for NSs within the Bunyaviridae family.

## INTRODUCTION

In 2011, a febrile syndrome was reported in adult dairy cows in Germany and the Netherlands and a novel virus, Schmallenberg virus (SBV), was discovered and found to be pathogenic for ruminants (1). This newly identified virus belongs to the Orthobunyavirus genus within the Bunyaviridae family. After its first emergence in Northern Europe, SBV rapidly spread across many European countries, causing a large epidemic (2). SBV predominantly affects domestic and wild ruminants and is transmitted by multiple species of Culicoides biting midges (3–6). In pregnant females, transplacental infection can lead to stillbirths and abortions or cause severe congenital malformations in calves, lambs, and goat kids (1, 7, 8).

It is well established that the bunyavirus-encoded NSs protein contributes to viral pathogenesis by inhibiting host cell transcription and consequently the innate antiviral response (9–13). The role of SBV NSs as a virulence factor has been investigated using an NSs deletion mutant (SBVΔNSs) produced by reverse genetics. In NIH-Swiss mice inoculated by intracerebral route, SBVΔNSs showed an attenuated phenotype characterized by a delay in the time of death in comparison to wild-type (WT) SBV (7). This shows that SBV NSs plays a major role in viral pathogenesis. SBVΔNSs, in contrast to its wild-type counterpart, is able to induce the synthesis of interferon (IFN) in several cell lines, demonstrating that SBV NSs inhibits the host IFN response (7, 14). Interestingly, SBV NSs is also able to trigger the proteasomal degradation of the Rpb1 subunit of RNA polymerase II (Pol II) *in vitro* and subsequently to inhibit cellular transcription and protein synthesis. The blockade of the IFN response by NSs may be a consequence of this global inhibition of transcription (15). Besides, a transcriptomic study has shown *in vitro* that SBV NSs causes a shutdown in the expression of genes involved in innate immunity. Nevertheless, this shutdown is incomplete since a few antiviral genes are still expressed following SBV infection (16). In addition, Barry et al. showed that SBV NSs could enhance the rate of apoptotic cell death (15).

In the present study, we identified a nucleolar localization signal (NoLS) between amino acids 33 and 51 (designated “aa 33–51” here) of SBV NSs that allows its colocalization with naturally resident nucleolar proteins, such as B23 (nucleophosmin) and fibrillarin. Most importantly, wild-type SBV induces nucleolus-to-nucleoplasm relocalization of B23 in several cell systems, including primary human neural progenitor cells (hNPCs). In contrast, the distribution of this protein was unmodified in cells infected with a mutant virus expressing an NSs variant lacking NoLS (SBVΔNoLS). We also show that an NSsΔNoLS mutant protein could not inhibit a cytomegalovirus (CMV)-driven promoter activity in comparison to its wild-type counterpart. To our knowledge, this is the first characterization of nucleolar targeting of a NSs protein from bunyaviruses.

## RESULTS

### SBV NSs subcellular localization.

**FIG 1:**
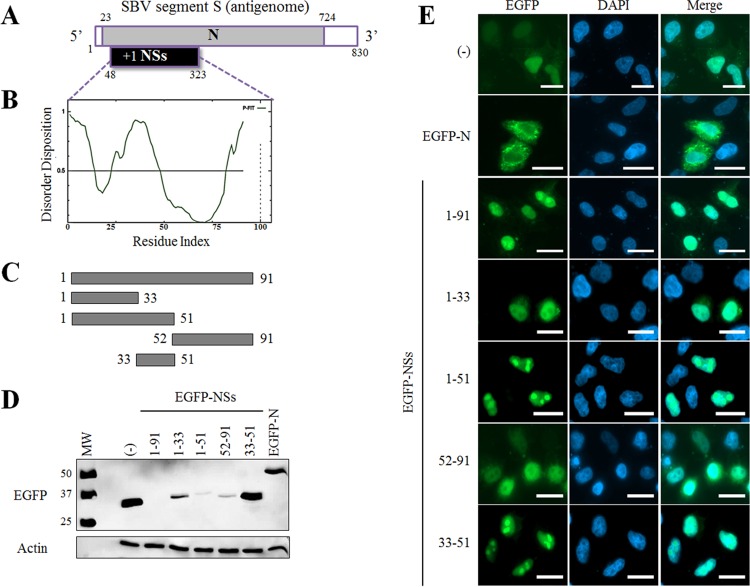
Expression and localization of SBV NSs mutants. (A) Schematic representation of SBV segment S (antigenomic sense). The 5′ and 3′ noncoding regions are indicated in white. The open reading frame (ORF) of the N protein is located between nucleotides 23 and 724. The NSs sequence is encoded by a +1 ORF between nucleotides 48 and 323. (B) Prediction of intrinsically disordered residues in SBV NSs using PONDR-FIT. The horizontal line at 0.5 of the *y* axis represents the threshold for disordered/structured residues. Residues with a score above this line are predicted to be disordered, and residues with a score below 0.5 are predicted to be ordered. (C) Schematic representation of NSs constructs used in panels D and E. (D) HEK 293T cells were transfected with 500 ng pEGFP-C1, pEGFP-N, or different pEGFP-NSs mutants as indicated. At 24 h posttransfection, cells were lysed, and lysates were used for detection of EGFP constructs by Western blot analysis. β-Actin was used as an internal control. (E) HeLa cells were transfected as in panel D. Twenty-four hours posttransfection, cells were fixed with 4% PFA and labeled with DAPI (blue) to stain nuclei. Intracellular localization of EGFP constructs (green) was visualized by fluorescence microscopy (×63 magnification). Scale bars represent 20 μm.

The S segment of SBV encodes the nucleoprotein N and the nonstructural protein NSs (Fig. 1A). NSs is encoded by an open reading frame (ORF) between nucleotides 48 and 323 of the antigenomic RNA in position +1 with respect to the ORF encoding the nucleoprotein N. NSs is poorly detected in SBV-infected cells or in cells transiently transfected with NSs expression plasmids (15; this work). This lack of expression might be partially attributed to an intrinsically unstable nature of the protein, which often relies on the presence of disordered domains. Indeed, *in silico* analyses performed with the PONDR-FIT software predict several natively disordered regions along the primary amino acid sequence (Fig. 1B). Especially, the N- and C-terminal ends of the protein as well as a central domain encompassing aa 33–51 appeared highly disordered, suggesting that NSs is an intrinsically unstructured protein. In order to assess the respective contribution of the ordered/disordered predicted regions in NSs expression and function, we generated expression plasmids for NSs and NSs deletion mutants containing or not the disordered sequence from aa 33 to aa 51 (Fig. 1C). We also cloned the full-length nucleoprotein (N) ORF as a control. All of these constructs were expressed as enhanced green fluorescent protein (EGFP) fusion proteins, and their expression was assessed by Western blotting (WB) upon transfection of HEK 293T cells using an anti-EGFP antibody (Fig. 1D). Full-length EGFP-NSs was mostly undetectable, although a very faint band could be inconstantly observed in some experiments, while all other NSs mutants and the N protein were detected in variable amounts. Then we assessed the intracellular distribution of these constructs in HeLa cells (Fig. 1E). As expected, EGFP alone displayed a diffuse pattern throughout the cell, whereas EGFP-N mainly localized in the cytoplasm, as previously described (15). All NSs constructs, including the previously poorly detected full-length EGFP-NSs, were mostly present in the nucleus of transfected cells. Among those, EGFP-NSs(1–51) and EGFP-NSs(33–51) proteins were observed in specific dense punctate structures that were evocative of a nucleolar distribution. On the contrary, EGFP-NSs(1–33) and EGFP-NSs(52–91) constructs which do not contain the aa 33–51 disordered sequence are excluded from these subnuclear structures. Similar distribution patterns were found when an ovine cell line and a bovine cell line were analyzed (data not shown).

### NSs contains a nucleolar localization signal (NoLS) and induces nucleolar disorganization.

**FIG 2:**
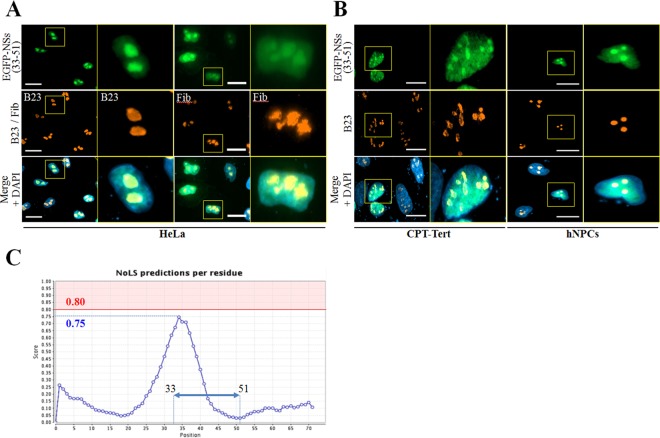
NSs contains a nucleolar targeting domain. (A and B) HeLa and CPT-Tert cells and hNPCs were transfected with 500 ng of pEGFP-NSs(33–51). At 24 h posttransfection, cells were fixed with 4% PFA and labeled with DAPI to stain nuclei and with specific antibodies for B23 and fibrillarin (Fib). Intracellular localization of DAPI-stained nuclei (blue), EGFP-NSs(33–51) (green), B23 and fibrillarin (red) was visualized by fluorescence microscopy (×63 magnification). One cell per picture was enlarged on the right side (yellow insets). Scale bars represent 20 μm. (C) Graphic representation showing the average NoLS prediction score in the SBV NSs protein using NoD, a nucleolar localization sequence detector. The horizontal red line at value 0.8 of the *y* axis represents the threshold for NoLS prediction. Amino acid sequences with a score above this line are NoLS candidate segment regions and represent the range of scores within which a 20-residue segment is predicted to be an NoLS. The blue dotted line represents the score reached by SBV NSs, and the blue double-headed arrow indicates the aa 33–51 peptide localization.

The atypical subnuclear localization of EGFP-NSs(33–51) prompted us to determine whether this chimeric protein was localized in nucleolar compartments. Thus, we carried out fluorescence microscopy in HeLa cells using the EGFP-NSs(33–51) construct and the nucleolus-resident proteins B23 and fibrillarin (Fig. 2A). EGFP-NSs(33–51) strongly colocalizes with both B23 and fibrillarin in transfected cells, indicating that this domain acts as a nucleolar targeting sequence. A similar distribution pattern was observed in ovine CPT-Tert cells and primary hNPCs (Fig. 2B). Then we searched for the presence of a nucleolar localization signal (NoLS) in the NSs protein using the web server NoD (http://www.compbio.dundee.ac.uk/nod) (17). Although no NoLS was recognized within NSs sequence, the graphical output of the NoLS score revealed a peak (a value of 0.75) corresponding to a central region of the protein that almost reached the positive predictive value (i.e., values of ≥0.80 considered positive) (Fig. 2C). Interestingly, this region encompasses the aa 33–51 sequence that was seen in Fig. 1E and 2A and -B to display nucleolar localization. These data demonstrate that the aa 33–51 domain of SBV NSs contains a functional NoLS.

**FIG 3:**
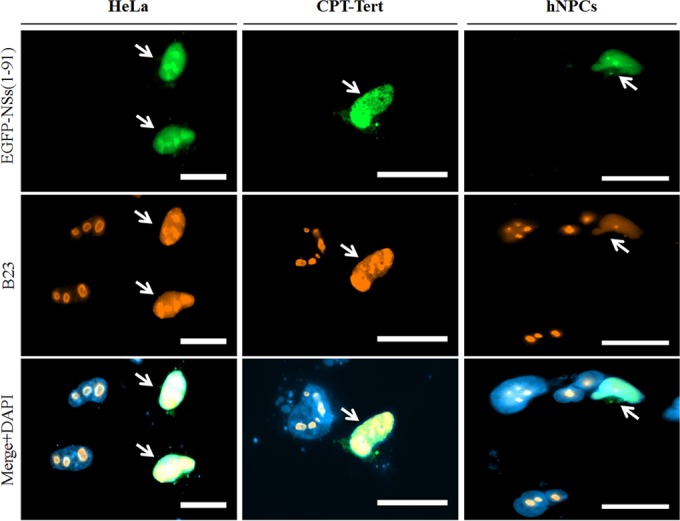
NSs triggers nucleolar reorganization. (A) HeLa and CPT-Tert cells and hNPCs were transfected with 500 ng of pEGFP-NSs(1–91). At 24 h posttransfection, cells were fixed with 4% PFA and labeled with DAPI to stain nuclei or stained with specific antibody for B23. Intracellular localization of DAPI-stained nuclei (blue), EGFP constructs (green), and B23 (red) was visualized by fluorescence microscopy (×63 magnification). Transfected cells are indicated using white arrows. Scale bars represent 20 μm.

In order to assess whether the full-length NSs is also present in the nucleolus, fluorescence microscopy was performed in HeLa and CPT-Tert cells and primary hNPCs transfected with the pEGFP-NSs(1–91) plasmid (Fig. 3). The full-length EGFP-NSs was predominantly observed in the nucleus of all cell types as described in the legend to Fig. 1, but nucleolar localization was only partly recognized. Interestingly, B23 was largely redistributed from the nucleolus to the nucleoplasm in cells expressing EGFP-NSs. Altogether these results suggest that, once targeted to the nucleolus through an internal NoLS motif, full-length NSs protein provokes nucleolar disorganization.

### SBV triggers nucleolar disruption at an early stage during infection.

**FIG 4:**
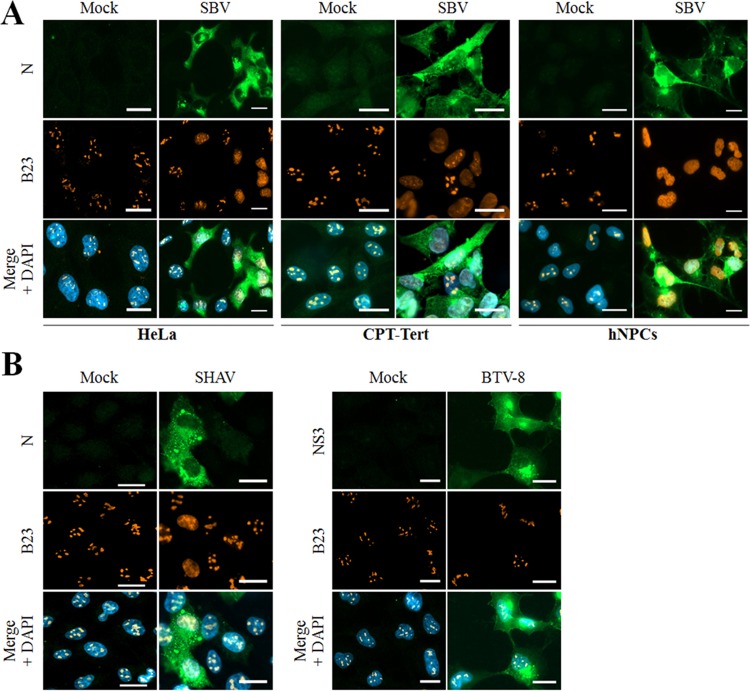
SBV and a Simbu-related virus trigger nucleolar reorganization during infection. (A) HeLa and CPT-Tert cells and hNPCs were mock infected or infected with the SBV WT (MOI of 0.01). After overnight incubation, cells were fixed with 4% PFA and labeled with DAPI to stain nuclei or stained with specific antibodies for B23 and SBV N. Intracellular localization of DAPI-stained nuclei (blue), B23 (red), and SBV N (green) was visualized by fluorescence microscopy (×63 magnification). Scale bars represent 20 μm. (B) HeLa cells were mock infected or infected with either Shamonda virus (SHAV [left panel]) or bluetongue virus (BTV [right panel]). After overnight incubation, cells were fixed with 4% PFA and labeled with DAPI to stain nuclei or stained with specific antibodies for B23, N SHAV, or NS3 BTV-8. Intracellular localization of DAPI-stained nuclei (blue), B23 (red), and SHAV N or BTV NS3 (green) was visualized by fluorescence microscopy (×63 magnification). Scale bars represent 20 μm.

To better understand whether SBV perturbs nucleolus organization during infection, CPT-Tert cells, hNPCs, and HeLa cells were infected with SBV (multiplicity of infection [MOI] of 0.01). Then the viral N protein and the nucleolus-resident protein B23 were detected at 20 h postinfection (p.i.) by fluorescence microscopy (Fig. 4A). In all SBV-infected cell types, B23 was fully redistributed throughout the nucleoplasm, while in mock-infected and neighbor-uninfected cells, B23 displayed its typical nucleolar pattern. An alteration of B23 localization was also induced by Shamonda virus (SHAV), while B23 distribution pattern was unmodified in bluetongue virus (BTV)-infected cells, despite the fact the NS4 protein of this virus localizes in the nucleolus (Fig. 4B). This suggests a putative conserved mechanism between bunyavirus members of the Simbu serogroup.

**FIG 5:**
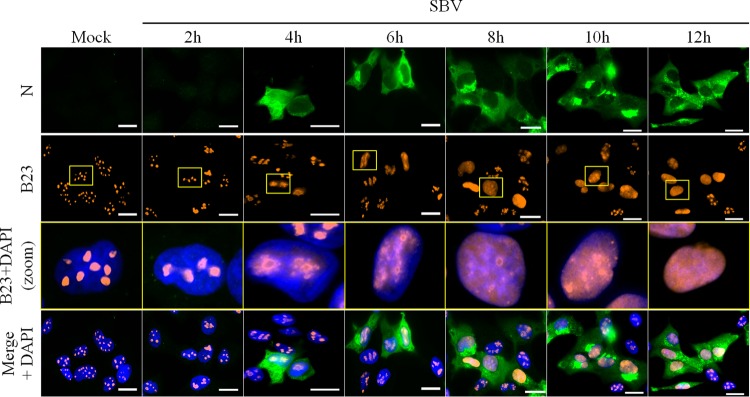
SBV-induced nucleolar reorganization occurs early during infection. CPT-Tert cells were infected with SBV WT (MOI of 0.1). At different time points p.i. (from 2 h to 12 h), cells were fixed with 4% PFA to study the subcellular localization of B23 and SBV N by fluorescence microscopy. After fixation, cells were labeled with DAPI to stain nuclei and stained with specific antibodies for B23 and SBV N. Intracellular localization of DAPI-stained nuclei (blue), B23 (red), and SBV N (green) was visualized by fluorescence microscopy (×63 magnification). One cell per picture was enlarged (yellow insets) to better assess gradual B23 relocalization. Scale bars represent 20 μm.

To determine the timing of B23 redistribution during SBV infection, CPT-Tert cells were mock infected or infected with SBV (MOI of 0.1) and fixed at different times p.i. (Fig. 5). As soon as 4 h p.i., nucleolar distribution of B23 was already moderately altered and was completely redistributed in the nucleoplasm of SBV-infected cells at 12 h p.i. Similar results were found when an MOI of 0.5 was used (data not shown). These data strongly suggest that reorganization of the nucleolus occurs early during SBV infection.

### SBV-induced nucleolar disorganization precedes Rpb1 degradation.

**FIG 6:**
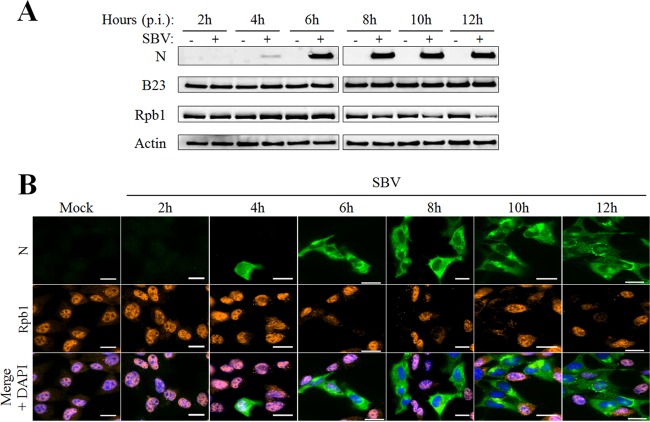
SBV-induced nucleolar reorganization occurs earlier than Rpb1 degradation. (A and B) CPT-Tert cells were infected with the SBV WT (MOI of 0.1). At different time points p.i. (from 2 h to 12 h), cells were either lysed for B23, Rpb1, SBV N and β-actin detection by Western blot analysis (A) or fixed with 4% PFA and subsequently labeled with DAPI to stain nuclei (blue) and stained with specific antibodies for Rbp1 (red) and SBV N (green). Scale bars represent 20 μm (B).

It has been shown recently that transcription inhibition observed during SBV infection results from the proteasomal degradation of the Rpb1 subunit of RNA Pol II, which started from 6 h p.i. and was maximal at 15 h p.i. (15). As nucleolar disorganization occurs early during SBV infection, we wondered if this event can precede Rpb1 degradation. To address this hypothesis, the expression of Rpb1 as well as the nucleolar protein B23 was first measured by Western blot analysis at different times p.i. as described in the legend to Fig. 5. In SBV-infected CPT-Tert cells, the Rpb1 expression level started to decrease from 8 h to 12 h p.i. in comparison with that in mock-infected cells (Fig. 6A), which is in accordance with previously published data (15). In contrast, the steady-state protein level of B23 was unmodified at all considered time points p.i., suggesting that SBV infection alters localization of B23 but not its expression.

Then we studied Rpb1 localization in SBV-infected cells at similar times p.i. (Fig. 6B). As expected, Rpb1 was found in the nucleus of mock-infected cells. In SBV-infected cells, Rpb1 labeling started to decrease at 6 h p.i. and was poorly detected or not detected at the subsequent time points p.i. The low proportion of SBV-infected cells at 6 h p.i. could explain the lack of Rpb1 decrease at this time point as measured by Western blotting. Altogether these results indicate that nucleolar reorganization probably occurs a few hours before Rpb1 degradation during SBV infection.

### Mutations in NoLS sequence impair NSs nucleolar localization.

**FIG 7:**
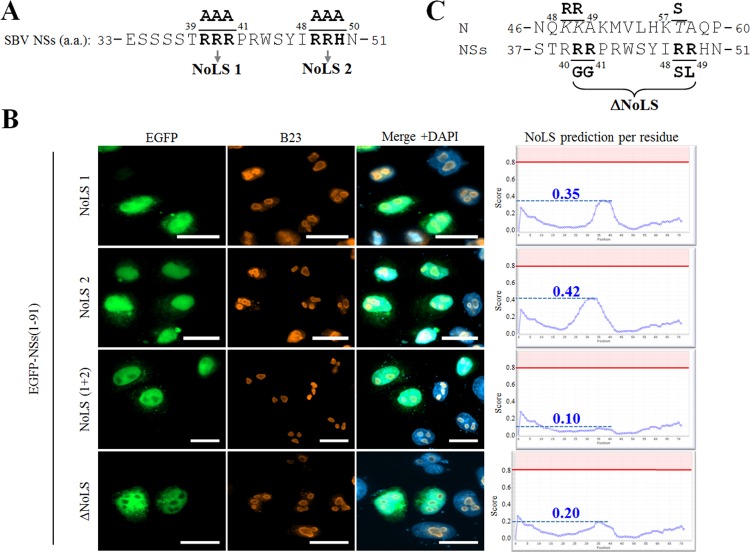
Mutation of a putative nucleolar localization signal impairs NSs nucleolar localization. (A) Amino acid sequence of the aa 33–51 region of SBV NSs. Bipartite triplet of basic residues corresponding to putative NoLS are depicted in bold type and termed NoLS 1 and NoLS 2. (B) HeLa cells were transfected with pEGFP-NSs(1–91) constructs mutated in NoLS 1, NoLS 2, both NoLS 1 and 2, and ΔNoLS as indicated. At 24 h posttransfection, cells were fixed with 4% PFA and labeled with DAPI to stain nuclei or stained with specific antibody for B23. Intracellular localizations of EGFP constructs (green), DAPI-stained nuclei (blue), and B23 (red) were visualized by fluorescence microscopy (×63 magnification). Scale bars represent 20 μm. Each corresponding NoD graphic (right) shows the average NoLS prediction score in the mutated SBV NSs protein. The horizontal red line at value 0.8 of the *y* axis represents the threshold for NoLS prediction, and the blue dotted line represents the score reached by mutated SBV NSs. (C) Amino acid sequence of the aa 37–51 region of NSs and aa 46–60 region of N. Minimal NoLS mutations designed to be introduced in a recombinant virus were termed ΔNoLS and are depicted in bold type under the NSs sequence. Modified amino acids in NSs sequence appear in bold type, and consecutive changes in N sequence are represented in italic above the NSs sequence.

We next sought to identify the critical residues of the NSs(33–51) domain involved in nucleolar targeting. NoLS motifs are often enriched in basic residues (18). Interestingly, the NSs(33–51) domain contains two close stretches of basic residues: RRR(39–41) and RRH(48–50) (Fig. 7A). Thus, we aimed to assess the role of these positively charged residues in nucleolar targeting by replacing them by alanine residues. Either the first (NoLS 1), the second (NoLS 2), or both (NoLS 1+2) basic stretches were modified within the pEGFP-NSs(1–91) plasmid construct. Cells transfected with either NoLS 1 or NoLS 2 mutants showed a predominant nuclear distribution with no or poor nucleolar localization, while the distribution of B23 appeared unaffected (Fig. 7B). Interestingly, the double NoLS 1+2 mutant displayed nuclear distribution, but nucleolar localization was completely abolished, suggesting that both positively charged sites are important for nucleolar targeting. These observations correlate with *in silico* analyses in which mutations of both basic stretches appeared to be required to fully impair NoLS prediction (<0.15) (Fig. 7B, right).

In order to specifically address the importance of NSs-induced nucleolar disorganization in virus replication cycle, we aimed to produce a recombinant virus expressing an NSs protein unable to reach the cell nucleolus. As N and NSs ORFs overlap on the S segment, nucleotide changes introduced in the NSs ORF could have a detrimental impact on N function and consequently on SBV replication. Computational predictions suggested that the last two residues of NoLS 1 and the first two residues of NoLS 2 were the most critical residues for nucleolar targeting. Based on this assumption, we designed an NSs mutant with predicted impaired nucleolar localization and low impact on N ORF (Fig. 7C). We replaced RR residues in positions 41 and 42 (NoLS 1) with GG residues and the RR residues in positions 48 and 49 (NoLS 2) with SL residues. While these substitutions created three nonsynonymous mutations in the N ORF (R48K, R49K, and T57S), the global charges and properties of the modified residues were preserved. Site-directed mutagenesis was used to introduce these mutations within pEGFP-NSs(1–91) to generate an EGFP-NSs(ΔNoLS) mutant. Fluorescence microscopy was then used to study the subcellular localization of EGFP-NSs(ΔNoLS) (Fig. 7B, lower panel). As predicted, EGFP-NSs(ΔNoLS) was found in the nucleus but not in the nucleolus, hence validating the suppression of NSs NoLS function.

### An SBV mutant lacking NoLS is impaired in nucleolar disruption.

**FIG 8:**
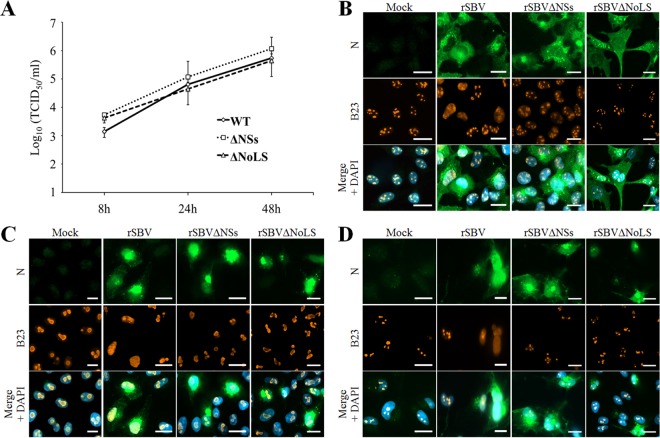
A SBVΔNoLS mutant virus does not induce nucleolar reorganization. (A) Replication kinetics of rSBV WT, rSBVΔNSs, and rSBVΔNoLS was assessed in CPT-Tert cells (MOI of 0.1). After infection, cell supernatants were collected from 8 h to 48 h p.i. and the viral titers were obtained by limiting dilution assays in CPT-Tert cells. Each experiment was performed in triplicate, and bars indicate the standard errors. (B to D) CPT-Tert cells (B), HeLa cells (C), and hNPCs (D) were mock infected or infected with rSBV WT, rSBVΔNSs, and rSBVΔNoLS (MOI of 0.01). After 24 h of infection, cells were fixed with 4% PFA and labeled with DAPI to stain nuclei or stained with specific antibody for B23. Intracellular localization of DAPI-stained nuclei (blue), B23 (red), and SBV N (green) was visualized by fluorescence microscopy (×63 magnification). Scale bars represent 20 μm.

We then generated a mutant virus, rSBVΔNoLS, harboring these mutations by reverse genetics. We also rescued a wild-type SBV (rSBV WT) and rSBVΔNSs, an SBV recombinant mutant virus lacking the entire NSs protein. rSBVΔNoLS displayed the same replication kinetics and reached the same titers as rSBV WT and rSBVΔNSs in IFN-deficient CPT-Tert cells (Fig. 8A).

Then the rSBVΔNoLS mutant was used to assess the impact of NSs localization on nucleolar organization during SBV infection. HeLa and CPT-Tert cells and hNPCs were mock infected or infected with rSBV WT, rSBVΔNSs, or rSBVΔNoLS (MOI of 0.01) (Fig. 8B to D). Cells were fixed at 18 h p.i., and B23 was detected by fluorescence microscopy. In mock-infected cells, B23 displayed a typical nucleolar pattern. In all cell types infected with rSBV WT, B23 was strongly redistributed throughout the nucleoplasm, while it was unchanged in neighbor-uninfected cells. However, we did not observe any redistribution of B23 in both rSBVΔNSs- and rSBVΔNoLS-infected-cells. Thus these data demonstrate that NSs impairs the B23 natural distribution during SBV infection and NSs nucleolar localization is necessary for this event.

### NSs-induced transcriptional shutoff activity is hampered with ΔNoLS mutants.

**FIG 9:**
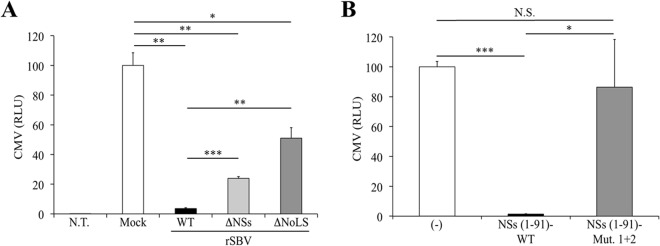
Involvement of the NoLS motif in NSs-induced cellular shutoff. (A) HEK 293T cells were not transfected (N.T.) or were transfected with 100 ng of pCMV-Luc(Firefly) reporter plasmid. After overnight incubation, cells were mock infected or infected with rSBV, rSBVΔNSs, or rSBVΔNoLS. Cell lysates were harvested 24 h p.i. and used to determine luciferase activities. Mean ratios of activities of triplicate samples were calculated and are presented as percentages of the mock control (±standard deviations). (B) HEK 293T cells were transfected with 250 ng of empty plasmid (−) or a plasmid encoding the 3×FLAG-NSs WT or NoLS 1+2 mutant together with 100 ng of the luciferase reporter plasmid pCMV-Luc(Firefly). Cell lysates were harvested 24 h after transfection and used to determine luciferase activities. Mean ratios of activities of triplicate samples were calculated and are presented as percentages of the control (±standard deviations). Results are representative of one experiment and were reproduced in three independent experiments. Statistically significant differences are indicated: *, *P* < 0.05; **, *P* < 0.01, and ***, *P* < 0.001, by Student's *t* test. N.S., not significant.

It has recently been shown that SBV NSs inhibits IFN synthesis, and as a consequence, an SBV recombinant virus lacking NSs is attenuated *in vivo* (7, 19). Moreover, Barry et al. found that NSs is able to block global cellular synthesis, including the antiviral response (15). To assess the role of nucleolar localization in the ability of SBV NSs to interfere with gene expression, HEK 293T cells were mock infected or infected with rSBV, rSBVΔNSs, or rSBVΔNoLS (MOI of 0.1) and transfected with a plasmid expressing firefly luciferase under the control of the CMV promoter, pCMV-Luc(Firefly). Luciferase activity was measured 24 h later (Fig. 9A). As expected, luciferase activity was strongly inhibited in rSBV-infected cells (>90% inhibition) compared to mock-infected cells. In contrast, luciferase activity was partly restored in cells infected with rSBVΔNoLS (49% inhibition) and to a lesser extent with rSBVΔNSs (74% inhibition). This suggests that the NSs nucleolar localization is also needed to counteract RNA Pol II-driven cellular transcription. Moreover, Rpb1 as well as cellular RNA levels were unmodified in rSBVΔNoLS-infected cells (data not shown). Then a similar experimental approach was used to assess the effect of an NSs NoLS mutant on gene expression. We transfected HEK 293T cells with pCMV-Luc(Firefly) and plasmids encoding WT NSs or mutant NoLS 1+2 (Fig. 9B). As expected, the NSs(1–91) WT drastically repressed the activation of the firefly luciferase gene expression driven by the CMV promoter. In contrast, luciferase activity was fully restored in cells transfected with the NSs NoLS 1+2 mutant. Collectively, these results suggest that gene expression shutoff exerted by SBV NSs might require its proper localization to the nucleolus.

## DISCUSSION

The NSs protein is a well-documented virulence factor within the Bunyaviridae that interferes with the cellular antiviral responses at the transcriptional level to facilitate viral replication and propagation. In particular, the Rpb1 subunit of RNA Pol II polymerase is targeted by several orthobunyaviruses, including the recently emerged SBV (9, 15, 20–22). In this study, we found that the NSs protein of SBV contains a nucleolar localization signal (NoLS) that is important for NSs function and disturbs the normal distribution of nucleolus-resident proteins. While it is well known that the majority of NSs proteins of bunyaviruses are nuclear proteins, to our knowledge, this is the first report of the presence of an NoLS within an NSs protein.

Until recently, the nucleolus was considered a subnuclear structure responsible for rRNA synthesis and nascent ribosome assembly. Several proteomic studies have shown that more than 6,000 resident or transitory proteins traffic through the nucleolus. The nucleolus is highly dynamic and plays fundamental roles in cellular stress responses, innate immune responses, or cell cycle regulation (reviewed in reference 23). Given the variety of key functions ensured by the nucleolus, many viruses that replicate in the nucleus (DNA viruses, retroviruses, and some negative-stranded RNA viruses) have usurped the nucleolus to favor their own replication (24–27). There are now many examples of viral proteins targeting the nucleolus, but the molecular function behind this localization is often uncertain or unresolved.

Most of the nucleolar proteins contain basic residue-rich sequences that act as nucleolar localization signals (NoLSs), although a consensus NoLS motif(s) has not been clearly defined. This is possibly due to the huge range of interactors involved in nucleolar localization. In this study, we found that the NSs region between amino acid residues 33 and 51 of NSs specifically targets this protein to the nucleolus and therefore acts as an NoLS. Fine dissection of this NoLS sequence allowed us to show that two stretches of basic residues are crucial for nucleolar targeting. Nucleolar localization of a protein is often governed by its association with another nucleolus-resident protein. Alternatively, the protein can traffic to the nucleolus on its own, where it associates with protein(s) and/or ribosomal DNA (rDNA)/rRNA to be retained in this compartment. NoLSs are often intimately associated with nuclear localization signals (NLSs) that are also enriched in basic residues and mediate active transport of proteins through the nuclear pore complex (28). We now aim to decipher the sequences involved in the nuclear import of NSs. We believe that NSs could traffic actively between the cytoplasm and the nucleus, while the NoLS functions more as a retention signal (unpublished data).

In our study, we found that SBV infection or NSs overexpression triggered the redistribution of B23 throughout the nucleoplasm. On the other hand, NoLS mutation in SBV NSs impaired NSs-induced transcription shutdown. These results raise the question about the link between nucleolar localization of NSs and transcription inhibition. Among the multiple strategies evolved by viruses to interfere with the host transcription machinery (29), nucleolar targeting is a common mechanism shared by several picornaviruses (24, 30). Indeed, the 3C^pro^ protease of poliovirus blocks RNA Pol I synthesis through modification and inactivation of factors essential for rRNA transcription, upstream binding factor (UBF) and selectivity factor 1 (SL1) possibly involving its proteolytic activity (31). The encephalomyocarditis virus encodes several proteins (2A, 3B^VPg^, 3C^pro^, and 3D^pol^) that also localize to the nucleolus at an early stage during infection (32). Although elucidation of the precise molecular mechanisms of the shutdown of cellular transcription by picornaviruses awaits further research, the nucleolus is clearly a primary interface in this process. In parallel, several studies showed that transcription inhibition can induce nucleolar disruption (reviewed in reference 33). Here, we found that nucleolar reorganization observed during SBV infection might occur slightly before Rpb1 degradation. However, we cannot rule out the possibility that RNA polymerase II activity might be block at an earlier time point. Thus, although the data presented in this article clearly show the requirement of an intact NoLS motif on NSs to ensure its intact subcellular localization and inhibitory function on RNA synthesis, further experiments are needed to decipher the exact molecular mechanisms involved in these events.

To date, only two other viral proteins have been reported to trigger Rpb1 degradation during infection: the NSs protein of the SBV-related orthobunyavirus La Crosse virus (LACV) (9) and the NSP2 protein of alphaviruses (34). This degradation seems to be linked to the blockade of cellular antiviral responses described for these proteins (9, 12, 35). Strikingly, the NSP2 protein encoded by the alphavirus Semliki Forest virus (SFV) is targeted to the nucleolus (36). Although the NoLS of SBV NSs is not fully conserved with LACV NSs, it would be interesting to assess whether the latter could also be addressed to the nucleolus through a specific sequence.

In an attempt to identify determinants of SBV virulence, Varela et al. serially performed extensive passage and plaque purification of SBV in CPT-Tert cells. They unexpectedly obtained an SBV mutant (SBVp32) with increased pathogenicity upon inoculation to newborn NIH-Swiss mice intracerebrally (7). SBVp32 accumulated 17 nucleotide changes among the three genome segments, with most of mutations identified within the M and S segments. By *in vivo* assays, they recently found that the M segment contains the determinants of virulence of SBVp32. In contrast, a reassortant containing the S segment of SBVp32 and the remaining genome segments from the SBV wild type is attenuated *in vivo*. Importantly, this naturally attenuated mutant harbors a nonsynonymous change at nucleotide 167, which induces a mutation in the first basic stretch of the NoLS motif, which is critical for nucleolar localization of NSs (37). The role of this natural *in vitro*-occurring mutation in SBV virulence has not specifically been addressed, but it underlines the importance of the NSs NoLS sequence in the SBV life cycle. Preliminary data suggest that the replication of the rSBVΔNoLS recombinant virus is impaired in IFN-competent cells, as previously observed with an SBVΔNSs mutant (7, 15), especially at late time points (data not shown). Thus, further work will be required to precise role of the NSs NoLS motif in the cellular antiviral response and in SBV attenuation.

Among the wide range of lesions that have been reported in SBV-infected fetuses and newborns, the central nervous system (CNS) constitutes a privileged target (7, 38–41). Indeed, loss of neural cells, and especially progenitor cells that give rise to precursors of neuronal and glial cell populations, is suspected to play a major role in SBV pathogenesis in domestic ruminants (E. Laloy, unpublished data). Whether cell loss either results from a direct SBV-driven cytopathic effect or is the consequence of an ischemic/inflammatory response requires further investigations. However, the presence of SBV antigens in neurons of associated lesions is in favor of the former hypothesis (7, 38, 39). Similarly Akabane virus is able to infect neuronal and astroglial cells, which led to degenerative death (42). In this study, we were able to demonstrate that SBV replicates efficiently in a model of primary human neural progenitor cells (hNPCs) and that NSs is highly efficient in these cells to relocate nucleolar proteins. It is interesting to note that recent findings point toward novel functions of the nucleolus in neuronal disorders (43, 44). This strengthens the putative link between NSs-triggered nucleolar alteration and SBV pathogenesis.

In conclusion, the present article clearly shows that the NSs protein of SBV is targeted to the nucleolus by an NoLS identified in the central region of the protein. The presence of NSs in the nucleolar compartment causes a nucleolar stress pointed out by the relocalization of nucleolar proteins. We cannot rule out that NSs possesses other biological functions outside the nucleolus, but we think that nucleolar stress is the main driver for NSs to prompt transcriptional shutoff and, therefore, provide the molecular basis explaining the role of SBV NSs as a virulence factor.

## MATERIALS AND METHODS

### Cells.

HeLa cells (kindly provided by Damien Arnoult, Inserm U1014, Hôpital Paul Brousse, Villejuif, France), HEK 293T cells, Vero cells (kindly provided by Pierre-Olivier Vidalain, UMR8601, CNRS-Université Paris-Descartes, Paris, France), and BSR-T7/5 cells (kindly provided by Jean-François Eleouët, INRA VIM, Jouy-en-Josas, France) were grown in Dulbecco's modified Eagle's medium (DMEM [Gibco]) supplemented with 10% heat-inactivated fetal calf serum (FCS [Eurobio]), and 1% sodium pyruvate with 100 IU/ml penicillin plus 100 μg/ml streptomycin (P-S [Gibco]). BSR T7/5 cells stably expressing the T7 polymerase were cultured in the presence of G418 (final concentration of 50 μg/ml) every five passages to maintain T7 expression. BHK-21 cells (kindly provided by the National Reference Laboratory for Orbiviruses, Laboratory for Animal Health, ANSES, Maisons-Alfort, France) were grown in Glasgow MEM (GMEM [Sigma-Aldrich]) supplemented with tryptose phosphate broth, 10% FCS, and P-S. The ovine CPT-Tert cell line (kindly provided by David Griffiths, Moredun Research Institute, Penicuik, UK) derives from choroid plexus cells (45) and was grown in Iscove's modified Dulbecco's medium (IMDM [Sigma-Aldrich]) supplemented with 10% FCS, 1% sodium pyruvate, 1 mM nonessential amino acids, and P-S. Human neural progenitor cell cultures (hNPCs) were prepared from human fetal brains (first trimester stage [see references 46 and 47 for details]). hNPCs were maintained in advanced DMEM–F-12 (Invitrogen Life Technologies) supplemented with l-glutamine (2 mM [Gibco]), apotransferrin (0.1 mg/ml [Sigma-Aldrich]), insulin (25 μg/ml [Sigma-Aldrich]), and progesterone (6.3 ng/ml [Sigma-Aldrich]). Medium was changed 3 times a week, and epidermal growth factor and basic fibroblast growth factor (both at 20 ng/ml [Abcys]) were added to maintain undifferentiated cells. All cells were cultured at 37°C in 95% air–5% CO_2_.

### Viruses and infections.

The wild-type SBV viral strain was isolated from the brain of a stillborn malformed lamb in the department of Moselle in France in 2012 (UMR1161 Virology Collection, ANSES, Maisons-Alfort, France). Virus titers were determined by standard plaque assays or endpoint dilution in CPT-Tert cells and expressed as PFU per milliliter or 50% tissue culture infective dose (TCID_50_) per milliliter. Shamonda virus (SHAV) was kindly provided by Philippe Despres (Institut Pasteur, France). The wild-type field strain of bluetongue virus serotype 8 (BTV-8) was isolated in the department of Ardennes in 2006 (48). Infections were performed with subconfluent HeLa and CPT-Tert cells or hNPCs. Cells were washed in serum-free medium and then inoculated with the viral inoculum at a multiplicity of infection (MOI) of 0.01 or 0.1/cell diluted in serum-free medium. Serum-free medium was used as an inoculum for mock-infected cells. After 1 h of incubation at 37°C while stirring, the inoculum was discarded, and fresh medium supplemented with 10% FCS was added to cell cultures. After infection, cells were analyzed at the indicated time points by fluorescence microscopy or Western blotting.

### Plasmids.

DNA sequences containing full-length open reading frames (ORFs) of SBV N, NSs(1–91), or NSs deletion mutants corresponding to aa 1 to 33, 1 to 51, 52 to 91, or 33 to 51 were amplified by PCR and cloned by *in vitro* recombination into pDONR207 (Gateway system; Invitrogen) as described in references 49 and 50). These SBV coding sequences were subsequently recombined into the pEGFP-C1 or pCINeo-3×FLAG modified vectors using the Gateway cloning procedure to generate fusion proteins downstream of EGFP (pEGFP-N/NSs constructs) or 3×FLAG (pF-NSs constructs), respectively (50). NoLS mutants on NSs full-length plasmid constructs were generated by site-directed mutagenesis following the manufacturer's instructions (QuikChange II site-directed mutagenesis [Agilent]). The pCMV-Luc(Firefly) luciferase reporter plasmid was kindly provided by Frédérick Arnaud (UMR754 INRA-UCBL-EPHE). The pUCSBVST7, pUCSBVMT7, pUCSBVLT7, and pUCSBVΔNSsT7 plasmids encoding antigenomic viral S, M, L, and SΔNSs segments, respectively, were used to generate recombinant wild-type (rSBV) and ΔNSs (rSBVΔNSs) viruses as described in reference 7. pUCSBVΔNoLST7 was obtained by introducing 4 codon changes in pUCSBVST7 by site-directed mutagenesis to abolish the NSs nucleolar localization signal (Fig. 7).

### Transfections.

All transfections were performed with JetPRIME (Polyplus) according to the manufacturer's instructions.

### Reverse genetics.

The recombinant rSBV WT, rSBVΔNSs, and rSBVΔNoLS viruses were generated using the SBV reverse-genetics protocols described in reference 7. Briefly, BSR-T7/5 cells were transfected with 750 μg of each plasmid carrying the antigenome. Supernatants were collected 5 days posttransfection, and the titers of the rescued viruses were determined by plaque assays in CPT-Tert cells.

### Virus replication kinetics.

To assess the replication kinetics of recombinant SBV viruses, 24-well plates were seeded with 1 × 10^5^ CPT-Tert cells/well, and the cells were infected at an MOI of 0.01. Cell supernatants were collected at several time points postinfection (p.i.) and used to estimate the presence of infectious viral particles by endpoint dilution performed in CPT-Tert cells.

### Computational analyses.

PONDR-FIT, a meta-predictor of intrinsically disordered residues, was used to predict the disordered region(s) in the NSs amino acid sequence (available at www.disprot.org) (51). Prediction of nucleolar localization sequences was performed using the web server NoD (available at: http://www.compbio.dundee.ac.uk/www-nod/) (17).

### Antibodies.

SBV infection was detected using a rabbit polyclonal antiserum raised against the SBV N protein expressed as a His-tagged recombinant protein from baculovirus in insect cell line Sf9 (Western blotting [WB], 1:1,000; immunofluorescence [IF], 1:500) (UMR1161 Virology, ANSES, Maisons-Alfort, France). N and NSs proteins expressed in fusion downstream of EGFP were detected using mouse monoclonal anti-GFP antibody (Roche Life Science; reference no. 11814460001 [WB, 1:2,000]). Monoclonal anti-B23 (reference no. B0556 [WB, 1:10,000; IF, 1:1,000]) and antiactin (clone AC-40; reference no. A3853 [WB 1:2,500]) antibodies were purchased from Sigma-Aldrich. Mouse monoclonal antibody against fibrillarin used in this study was from Abcam (IF, 1:250). Mouse anti-Rpb1 monoclonal antibody was purchased from Ozyme (clone 8WG16; reference no. BLE92010 [WB, 1:500; IF, 1:250]).

### Luciferase reporter assays.

The effect of NoLS mutations on NSs-driven inhibition of gene expression was assessed using luciferase reporter assays. Briefly, 7 × 10^5^ HEK 293T cells were transfected with 250 ng of empty plasmid (−) or plasmids encoding 3×FLAG-NSs WT or the NoLS 1+2 mutant together with 100 ng of the luciferase reporter plasmid pCMV-Luc(Firefly). Cells were incubated for 24 h and then lysed in luciferase lysis buffer (25 mM Tris [pH 7.8], 0.8 mM MgCl_2_, 0.1% Triton X-100, 15% glycerol). Firefly luciferase activity was determined using the Bright-Glo luciferase assay system (Promega). Each experiment was performed in triplicate at least three times independently. Mean ratios of activities of triplicate samples were calculated and presented as percentages of the control (±standard deviations). To assess the effect of SBV mutant viruses on the CMV promoter, 24-well plates were seeded with 7 × 10^5^ HEK 293T cells, and the cells were mock infected or infected with rSBV WT, SBVΔNSs, and SBVΔNoLS (MOI of 0.1). Immediately after infection, cells were transfected with 100 ng of pCMV-Luc(Firefly) and analyzed as described above.

### Immunofluorescence assays.

Twelve-millimeter-diameter coverslips in 24-well plates were seeded with CPT-Tert (2 × 10^5^) or HeLa (1 × 10^5^) cells. Twenty-four-well glass plates (Ibidi μ-plates, BioValley) precoated with Matrigel (1:1,000 [Corning, France]) were directly seeded with hNPCs (1 × 10^5^). After transfection and/or infection, cells were washed 3 times in phosphate-buffered saline (PBS) and fixed with 4% paraformaldehyde (PFA [Electron Microscopy Sciences]) in PBS for 20 min at room temperature. Cells were permeabilized with 0.5% Triton X-100 in PBS and incubated in blocking buffer (1% bovine serum albumin in PBS). The appropriate dilution of primary antibodies was then added for 1 h at room temperature. Cells were then washed three times in PBS, and Alexa Fluor 488 anti-mouse and Alexa Fluor 555 anti-rabbit secondary antibodies (Molecular Probes) were used to detect bound primary antibodies. Samples were mounted in Mowiol containing DAPI (4,6′-diamidino-2-phenylindole) for staining of nuclei (Sigma-Aldrich). Microscopy studies were carried out with an Axio observer Z1 fluorescence inverted microscope (Zeiss), and images were acquired using the Zen 2012 software and analyzed with ImageJ. Each experiment was reproduced at least 3 times.

### Western blot analysis.

Cells were harvested in lysis buffer (20 mM Tris-HCl [pH 7.5], 150 mM NaCl, 1% Triton X-100, and 10% glycerol supplemented with a cocktail of protease inhibitors according to the manufacturer's instructions (Complete EDTA-free [Roche Life Science]). Insoluble material was centrifuged at 10,000 × *g* for 15 min at 4°C, and the supernatant was kept as the soluble fraction. The total protein concentration of the soluble fraction was determined by a micro-bicinchoninic acid (micro-BCA) assay (Thermo Fisher). Protein extracts were reduced by being heated at 95°C for 5 min in the presence of denaturing agent, and an equal amount of proteins was resolved by 10% to 15% sodium dodecyl sulfate-polyacrylamide gel electrophoresis (SDS-PAGE), followed by transfer to nitrocellulose membrane (Hybond-ECL; Amersham Biosciences). Membranes were blocked with phosphate-buffered saline (PBS) containing 5% dry milk and 0.1% Tween 20 and incubated with the required dilution of specific antibodies. Bound primary antibodies were detected using horseradish peroxidase-conjugated anti-rabbit or anti-mouse secondary antibodies (Dako [diluted 1:5,000]) and an enhanced luminol-based chemiluminescent detection system (ECL clarity; Bio-Rad).

### Statistical analyses.

Statistical analyses were conducted with the Student *t* test. Differences were considered to be significant if the *P* value was <0.05.
